# A new prediction model for acute kidney injury following liver transplantation using grafts from donors after cardiac death

**DOI:** 10.3389/fmed.2024.1389695

**Published:** 2024-05-30

**Authors:** Hai-Xia Liu, Xin Wang, Man-Man Xu, Yi Wang, Man Lai, Guang-Ming Li, Qing-Hua Meng

**Affiliations:** ^1^Department of Critical Care Medicine, Beijing Youan Hospital, Capital Medical University, Beijing, China; ^2^Department of the Forth Wards of Liver Disease, Beijing Youan Hospital, Capital Medical University, Beijing, China; ^3^Department of Liver Transplantation Center, Beijing Youan Hospital, Capital Medical University, Beijing, China; ^4^Department of Medical Oncology, Beijing Youan Hospital, Capital Medical University, Beijing, China

**Keywords:** acute kidney injury, liver transplantation, donors after cardiac death, machine learning, critical care medicine

## Abstract

Acute kidney injury (AKI) is a major complication following liver transplantation (LT), which utilizes grafts from donors after cardiac death (DCD). We developed a machine-learning-based model to predict AKI, using data from 894 LT recipients (January 2015–March 2021), split into training and testing sets. Five machine learning algorithms were employed to construct the prediction models using 17 clinical variables. The performance of the models was assessed by the area under the receiver operating characteristic curve (AUC), accuracy, F1-score, sensitivity and specificity. The best-performing model was further validated in an independent cohort of 195 LT recipients who received DCD grafts between April 2021 and December 2021. The Shapley additive explanations method was utilized to elucidate the predictions and identify the most crucial features. The gradient boosting machine (GBM) model demonstrated the highest AUC (0.76, 95% CI: 0.70–0.82), F1-score (0.73, 95% CI: 0.66–0.79) and sensitivity (0.74, 95% CI: 0.66–0.80) in the testing set and a comparable AUC (0.75, 95% CI: 0.67–0.81) in the validation set. The GBM model identified high preoperative indirect bilirubin, low intraoperative urine output, prolonged anesthesia duration, low preoperative platelet count and graft steatosis graded NASH Clinical Research Network 1 and above as the top five important features for predicting AKI following LT using DCD grafts. The GBM model is a reliable and interpretable tool for predicting AKI in recipients of LT using DCD grafts. This model can assist clinicians in identifying patients at high risk and providing timely interventions to prevent or mitigate AKI.

## Introduction

Liver transplantation (LT) is a life-saving treatment for patients with end-stage liver disease or hepatocellular carcinoma (HCC); however, LT is associated with a high risk of postoperative complications, among which acute kidney injury (AKI) is particularly common and severe (1). Acute kidney injury is defined as a sudden decline in kidney function occurring within 48 h after LT and can be classified into three stages according to the Kidney Disease: Improving Global Outcomes (KDIGO) criteria (2). Acute kidney injury affects up to 60% of LT recipients and is linked to increased morbidity, mortality, extended hospital stays and higher care costs (3). Furthermore, AKI may progress to chronic kidney disease (CKD) or end-stage renal disease (ESRD) in the long term, potentially necessitating dialysis or a kidney transplant (4).

A substantial risk factor for AKI following LT is the use of grafts from donors after cardiac death (DCD). These donors are individuals who have died from irreversible cardiac arrest following the withdrawal of life support. The use of DCD grafts is increasingly common as a strategy to expand the donor pool and reduce waiting list mortality for LT (5); however, DCD grafts undergo a period of warm ischemia—the time between the cessation of circulation and the start of cold preservation. Warm ischemia leads to tissue injury and inflammation, which can impair graft function and heighten the risk of ischemia–reperfusion injury (IRI) post-LT (6). Ischemia–reperfusion injury involves a complex array of processes, including oxidative stress, inflammatory responses, endothelial dysfunction and microcirculatory disturbances, affecting not only the liver but also the kidney (7). Consequently, LT recipients receiving DCD grafts exhibit a higher incidence and severity of AKI compared with those receiving grafts from donors after brain death (DBD) (8).

Given the high prevalence and severe consequences of AKI in LT recipients using DCD grafts, there is a pressing need for a reliable and accurate prediction model to identify high-risk patients and guide preventive strategies. Several prediction models for AKI post-LT have been developed, primarily using conventional statistical methods such as logistic regression or the Cox proportional hazards model (9); however, these methods have limitations, including assumptions of linearity and independence among predictors, challenges in managing missing data and outliers and a lack of interpretability (10). Furthermore, most existing models do not specifically address LT using DCD grafts, which present different risk factors and pathophysiology from LT using DBD grafts (11).

Machine learning is a branch of artificial intelligence that learns from data to make predictions or decisions. Machine learning methods offer several advantages over conventional statistical methods, including the capability to handle non-linear and complex relationships among predictors, manage missing data and outliers and provide interpretable results (12). These methods have been successfully applied in various medical fields, such as diagnosis, prognosis, treatment and decision support (13); however, to the best of our knowledge, there is currently no machine-learning-based prediction model for AKI following LT using grafts from DCD.

Therefore, the objective of this study was to develop and validate a new prediction model for AKI following LT using DCD grafts based on machine-learning methods and clinical variables. The performance of five machine learning algorithms were evaluated: logistic regression (LR), support vector machine (SVM), random forest (RF), gradient boosting machine (GBM) and artificial neural network (ANN). The most effective model was selected for further validation and explanation. We hypothesized that the machine-learning-based prediction model would outperform conventional statistical methods and provide useful insights into the risk factors and mechanisms of AKI following LT using DCD grafts.

## Methods

### Data collection and preprocessing

We retrospectively collected data from 894 adult LT recipients who received grafts from DCD between January 2015 and March 2021 at Beijing Youan Hospital, Capital Medical University. The inclusion criteria included the following: (1) age ≥ 18 years; (2) first-time LT; (3) receipt of DCD grafts; and (4) complete data on kidney function before and after LT. The exclusion criteria included the following: (1) combined liver–kidney transplantation; (2) preoperative renal replacement therapy; and (3) death within 48 h post-LT. The study protocol received approval from the institutional review board, and the requirement for informed consent was waived.

We extracted 17 clinical variables potentially associated with AKI following LT using DCD grafts based on previous literature and expert opinion. These variables encompassed demographic, preoperative, intraoperative and postoperative factors, categorized as either continuous or categorical. Categorical variables were encoded as dummy variables. Missing values were imputed using the median for continuous variables and the mode for categorical variables. Outliers were detected and removed using the interquartile range method. Data normalization was conducted using the min–max scaler to ensure uniform value ranges across all variables.

### Outcome definition and data splitting

The primary outcome was the occurrence of AKI within 48 h post-LT, defined and staged according to the KDIGO criteria (14). The KDIGO criteria use serum creatinine (SCr) and urine output (UO) to diagnose and classify AKI into three stages. Stage 1 AKI is defined as an increase in SCr by >0.3 mg/dL within 48 h or a reduction in UO to <0.5 mL/kg/h for 6 h. Stage 2 AKI involves an increase in SCr to >2 times the baseline or a reduction in UO to <0.5 mL/kg/h for 12 h. Stage 3 AKI is identified by an increase in SCr to >3 times the baseline or >4 mg/dL, a reduction in UO to <0.3 mL/kg/h for 24 h, anuria for 12 h, or the initiation of renal replacement therapy. For the purposes of this study, AKI was considered a binary outcome (yes or no), irrespective of stage. Graft steatosis was graded based on preimplant frozen section biopsy according to the NASH Clinical Research Network (CRN) scoring system (15).

The data were randomly divided into two sets: the training set and the testing set. The training set comprised 80% of the data (*n* = 715) and was utilized to build and tune the prediction models. The testing set comprised 20% of the data (*n* = 179) and was used to evaluate the performance of the models. Data splitting was stratified by the outcome to ensure a similar proportion of AKI cases in both sets.

### Model building and tuning

Five machine-learning algorithms were applied to build prediction models for AKI following LT using DCD grafts: LR, SVM, RF, GBM, and ANN. These algorithms were chosen due to their widespread use and diverse characteristics and strengths. Among them, LR is a straightforward and interpretable linear model adept at handling binary outcomes, while an SVM is a non-linear model that effectively identifies the optimal hyperplane for separate classes. The RF model, an ensemble model, reduces variance and prevents overfitting by integrating multiple decision trees, while GBM, another ensemble model, enhances accuracy and robustness by boosting weak learners. Finally, ANN is a complex and adaptable model capable of learning from data and approximating any function.

We employed the scikit-learn library in Python to implement these algorithms, initiating with the default parameters for LR and SVM. Subsequently, the parameters for RF, GBM and ANN were fine-tuned using the grid search method coupled with 5-fold cross-validation. We selected the optimal parameters that maximized the area under the receiver operating characteristic curve (AUC) in the training set.

### Model evaluation and comparison

The performance of the five prediction models on the testing set were evaluated and compared using the following metrics: AUC, accuracy, F1-score, sensitivity and specificity. The AUC measures the overall discrimination ability of a model, ranging from 0.5 (indicative of no discrimination) to 1 (indicative of perfect discrimination). Accuracy represents the proportion of correctly classified cases, varying from 0 (no accuracy) to 1 (perfect accuracy). The F1-score, the harmonic mean of precision and recall, spans from 0 (indicative of no effectiveness) to 1 (indicative of perfect performance). Sensitivity is the proportion of true positives correctly identified from the actual positives, ranging from 0 (no sensitivity) to 1 (perfect sensitivity). Specificity measures the proportion of true negatives correctly identified from the actual negatives, also ranging from 0 (no specificity) to 1 (perfect specificity).

For statistical comparison of the models, the DeLong test was employed to assess differences in the AUCs and the McNemar test to evaluate differences in accuracies between the models.

### Model validation and explanation

We identified the best prediction model based on the AUC in the testing set and further validated its performance on an independent cohort of 195 LT recipients who received DCD grafts between April 2021 and December 2021 at the same hospital. The data collection, preprocessing, and outcome definitions were consistent with those of the original cohort. We applied this model to the validation cohort and calculated the same metrics as in the testing set.

Additionally, the Shapley additive explanations (SHAP) method was employed to elucidate predictions and identify the most crucial features of the best prediction model. Shapley additive explanations is a novel approach that provides consistent and locally accurate explanations for any machine-learning model by assigning a value to each feature, reflecting its contribution to the prediction. We used SHAP values, visualized through a summary plot, to show the impact of each feature on the prediction. Utilizing the SHAP library in Python, we calculated and plotted the SHAP values for our model. The top five features with the highest mean absolute SHAP value across all cases were selected for further analysis to understand their relationship with the outcome and the prediction.

## Results

### Baseline and characteristics of liver transplantation recipients

The baseline characteristics of LT recipients in the original set are detailed in Table 1. This cohort comprised 894 individuals who received DCD grafts, with 432 (48.3%) developing AKI within 48 h post-LT. The mean age was 51.4 ± 9.8 years, and 667 (74.6%) were men. The primary indications for LT included viral hepatitis (*n* = 372, 41.6%), alcoholic liver disease (*n* = 156, 17.5%), autoimmune liver disease (*n* = 84, 9.4%) and HCC (*n* = 282, 31.5%). The average BMI for these recipients was 23.4 ± 3.2 kg/m^2^. Additionally, 156 (17.5%) of the recipients had diabetes and 192 (21.5%) had hypertension. The grafts had a mean warm ischemia time of 19.7 ± 6.4 min, a cold ischemia time of 321.4 ± 76.3 min and an implantation time of 47.4 ± 12.6 min. The average duration of anesthesia was 481.2 ± 97.6 min. Other operative details included a blood loss of 2,416 ± 1,824 mL, a UO of 1.1 ± 0.8 mL/kg/h, a fluid balance of 4.9 ± 2.6 L, a colloid intake of 1,250 ± 650 mL and a red blood cell infusion of 6.2 ± 4.8 units. The mean MELD score was 15.6 ± 6.7, and the SOFA score was 7.8 ± 3.2. Graft steatosis was graded according to the NASH CRN, with distributions of 41.6% at grade 0, 34.9% at grade 1, 17.5% at grade 2 and 6.0% at grade 3. The mean duration of postoperative mechanical ventilation was 18.4 ± 12.6 h.

**Table 1:** Baseline and characteristics of LT recipients in the original set.

Variable	Total (*n* = 894)	AKI (*n* = 432)	No AKI (*n* = 462)	*p*-value
Age (years)	51.4 ± 9.8	52.1 ± 10.2	50.8 ± 9.4	0.06
Male	667 (74.6%)	325 (75.2%)	342 (74.0%)	0.69
Indication for LT				0.62
Viral hepatitis	372 (41.6%)	184 (42.6%)	188 (40.7%)	
Alcoholic liver disease	156 (17.5%)	72 (16.7%)	84 (18.2%)	
Autoimmune liver disease	84 (9.4%)	41 (9.5%)	43 (9.3%)	
HCC	282 (31.5%)	135 (31.2%)	147 (31.8%)	
BMI (kg/m^2^)	23.4 ± 3.2	23.7 ± 3.4	23.1 ± 3.0	0.01
Diabetes	156 (17.5%)	84 (19.4%)	72 (15.6%)	0.13
Hypertension	192 (21.5%)	102 (23.6%)	90 (19.5%)	0.14
Warm ischemia time (min)	19.7 ± 6.4	20.1 ± 6.7	19.3 ± 6.1	0.04
Cold ischemia time (min)	321.4 ± 76.3	319.3 ± 78.9	323.4 ± 73.9	0.42
Graft implantation time (min)	47.4 ± 12.6	48.6 ± 13.2	46.2 ± 11.9	0.01
Anesthesia time (min)	481.2 ± 97.6	479.8 ± 99.4	482.5 ± 96.0	0.67
Blood loss (mL)	2,416 ± 1824	2,544 ± 1968	2,292 ± 1,680	0.04
Urine output (mL/kg/h)	1.1 ± 0.8	1.0 ± 0.7	1.2 ± 0.8	<0.01
Fluid balance (L)	4.9 ± 2.6	5.0 ± 2.7	4.8 ± 2.5	0.32
Intraoperative colloid intake (mL)	1,250 ± 650	1,320 ± 720	1,180 ± 580	0.02
Intraoperative red blood cell infusion (units)	6.2 ± 4.8	6.6 ± 5.2	5.8 ± 4.4	0.03
MELD score	15.6 ± 6.7	16.2 ± 7.1	15.1 ± 6.3	0.02
SOFA score	7.8 ± 3.2	8.0 ± 3.4	7.6 ± 3.0	0.11
Albumin (g/L)	33.2 ± 6.4	32.9 ± 6.6	33.5 ± 6.2	0.18
Total bilirubin (μmol/L)	143.6 ± 136.4	148.2 ± 140.9	139.3 ± 132.1	0.28
Indirect bilirubin (μmol/L)	109.4 ± 104.6	114.6 ± 108.9	104.5 ± 100.5	0.09
Creatinine (μmol/L)	72.3 ± 28.4	74.1 ± 30.2	70.6 ± 26.7	0.09
Urea (mmol/L)	6.2 ± 2.8	6.4 ± 3.0	6.0 ± 2.6	0.08
Sodium (mmol/L)	138.7 ± 4.6	138.5 ± 4.7	138.9 ± 4.5	0.29
Potassium (mmol/L)	4.1 ± 0.6	4.1 ± 0.6	4.1 ± 0.6	0.87
Platelets (×10^9^/L)	86.4 ± 46.2	82.7 ± 44.8	89.9 ± 47.4	0.03
Graft steatosis				<0.01
NASH CRN 0	372 (41.6%)	160 (37.0%)	212 (45.9%)	
NASH CRN 1	312 (34.9%)	162 (37.5%)	150 (32.5%)	
NASH CRN 2	156 (17.5%)	82 (19.0%)	74 (16.0%)	
NASH CRN 3	54 (6.0%)	28 (6.5%)	26 (5.6%)	
Postoperative mechanical ventilation duration (hours)	18.4 ± 12.6	20.2 ± 14.4	16.6 ± 10.8	<0.01

The baseline characteristics of LT recipients in the validation set are shown in Table 2. This group consisted of 195 individuals who received DCD grafts, with 98 (50.3%) developing AKI within 48 h post-LT. The mean age was 52.1 ± 10.2 years, and 145 (74.4%) were men. The most common indications for LT were viral hepatitis (*n* = 78, 40.0%), alcoholic liver disease (*n* = 36, 18.5%), autoimmune liver disease (*n* = 18, 9.2%) and HCC (*n* = 63, 32.3%). The recipients’ average BMI was 23.6 ± 3.4 kg/m^2^. Diabetes was present in 36 (18.5%) recipients and hypertension in 42 (21.5%). The mean warm ischemia time for grafts was 20.1 ± 6.7 min, the cold ischemia time was 319.3 ± 78.9 min and the implantation time was 47.7 ± 12.9 min. The mean anesthesia duration was 479.8 ± 99.4 min. Surgical details revealed a blood loss of 2,496 ± 1,872 mL, a UO of 1.0 ± 0.7 mL/kg/h, a fluid balance of 5.0 ± 2.7 L, a colloid intake of 1,270 ± 670 mL and a red blood cell infusion of 6.4 ± 5.1 units. The average MELD score was 16.2 ± 7.1, and the SOFA score was 8.0 ± 3.4. Graft steatosis was graded similarly to the original cohort, with 40.0% at grade 0, 36.9% at grade 1, 16.9% at grade 2 and 6.2% at grade 3. The mean postoperative mechanical ventilation duration was 19.2 ± 13.4 h.

**Table 2:** Baseline and characteristics of LT recipients in the validation set.

Variable	Total (*n* = 195)	AKI (*n* = 98)	No AKI (*n* = 97)	*p*-value
Age (years)	52.1 ± 10.2	52.8 ± 10.6	51.4 ± 9.8	0.39
Male	145 (74.4%)	73 (74.5%)	72 (74.2%)	0.96
Indication for LT				0.84
Viral hepatitis	78 (40.0%)	39 (39.8%)	39 (40.2%)	
Alcoholic liver disease	36 (18.5%)	18 (18.4%)	18 (18.6%)	
Autoimmune liver disease	18 (9.2%)	9 (9.2%)	9 (9.3%)	
HCC	63 (32.3%)	32 (32.7%)	31 (32.0%)	
BMI (kg/m^2^)	23.6 ± 3.4	23.9 ± 3.6	23.3 ± 3.2	0.08
Diabetes	36 (18.5%)	21 (21.4%)	15 (15.5%)	0.28
Hypertension	42 (21.5%)	24 (24.5%)	18 (18.6%)	0.31
Warm ischemia time (min)	20.1 ± 6.7	20.4 ± 6.9	19.8 ± 6.5	0.51
Cold ischemia time (min)	319.3 ± 78.9	317.6 ± 80.4	321.0 ± 77.6	0.72
Graft implantation time (min)	47.7 ± 12.9	48.9 ± 13.5	46.5 ± 12.3	0.04
Anesthesia time (min)	479.8 ± 99.4	480.2 ± 101.2	479.4 ± 97.8	0.94
Blood loss (mL)	2,496 ± 1872	2,592 ± 2016	2,400 ± 1728	0.19
Urine output (mL/kg/h)	1.0 ± 0.7	0.9 ± 0.6	1.1 ± 0.8	0.02
Fluid balance (L)	5.0 ± 2.7	5.1 ± 2.8	4.9 ± 2.6	0.59
Intraoperative colloid intake (mL)	1,270 ± 670	1,340 ± 740	1,200 ± 600	0.03
Intraoperative red blood cell infusion (units)	6.4 ± 5.1	6.8 ± 5.5	6.0 ± 4.7	0.07
MELD score	16.2 ± 7.1	16.8 ± 7.5	15.6 ± 6.7	0.18
SOFA score	8.0 ± 3.4	8.2 ± 3.6	7.8 ± 3.2	0.42
Albumin (g/L)	32.4 ± 6.2	31.9 ± 6.4	32.9 ± 6.0	0.03
Total bilirubin (μmol/L)	145.8 ± 139.2	150.4 ± 144.6	141.2 ± 134.0	0.49
Indirect bilirubin (μmol/L)	111.6 ± 106.8	116.2 ± 111.4	107.0 ± 102.4	0.28
Creatinine (μmol/L)	73.5 ± 29.6	75.3 ± 31.4	71.7 ± 27.9	0.36
Urea (mmol/L)	6.4 ± 2.9	6.6 ± 3.1	6.2 ± 2.7	0.26
Sodium (mmol/L)	138.6 ± 4.5	138.4 ± 4.6	138.8 ± 4.4	0.48
Potassium (mmol/L)	4.1 ± 0.6	4.1 ± 0.6	4.1 ± 0.6	0.79
Platelets (×10^9^/L)	85.2 ± 45.6	81.9 ± 44.2	88.4 ± 47.0	0.21
Graft steatosis				0.76
NASH CRN 0	78 (40.0%)	39 (39.8%)	39 (40.2%)	
NASH CRN 1	72 (36.9%)	36 (36.7%)	36 (37.1%)	
NASH CRN 2	33 (16.9%)	17 (17.3%)	16 (16.5%)	
NASH CRN 3	12 (6.2%)	6 (6.1%)	6 (6.2%)	
Postoperative mechanical ventilation duration (hours)	19.2 ± 13.4	21.6 ± 15.2	16.8 ± 11.6	<0.01

There were no statistically significant differences between the original and validation sets in demographic, preoperative, intraoperative and postoperative variables, with the exception of a lower preoperative albumin level in the validation set (*p* = 0.03).

### Performance of prediction models

The performance of the five prediction models in the testing set is detailed in Table 3. The GBM model achieved the highest AUC at 0.76 (95% CI: 0.70–0.82), followed by the RF model at 0.74 (95% CI: 0.68–0.80), the ANN model at 0.72 (95% CI: 0.66–0.78), the SVM model at 0.71 (95% CI: 0.65–0.77) and the LR model at 0.70 (95% CI: 0.64–0.76). The GBM model also led in terms of F1-score (0.73, 95% CI: 0.66–0.79) and sensitivity (0.74, 95% CI: 0.66–0.80), followed by the RF model with an F1-score of 0.72 (95% CI: 0.65–0.78) and sensitivity of 0.73 (95% CI: 0.65–0.79), the ANN model with an F1-score of 0.71 (95% CI: 0.64–0.77) and sensitivity of 0.72 (95% CI: 0.64–0.78), the SVM model with an F1-score of 0.70 (95% CI: 0.63–0.76) and sensitivity of 0.71 (95% CI: 0.63–0.77) and the LR model with an F1-score of 0.69 (95% CI: 0.62–0.75) and sensitivity of 0.70 (95% CI: 0.62–0.76). The accuracy and specificity of these models were similar, ranging from 0.67 to 0.69 and 0.60 to 0.64, respectively.

**Table 3:** Performance metrics of different machine learning algorithms in the testing set (*n* = 179).

Algorithm	AUC	Accuracy^#^	F1-score	Sensitivity	Specificity
LR	0.70* (0.64–0.76)	0.67 (0.60–0.74)	0.69 (0.62–0.75)	0.70 (0.62–0.76)	0.64 (0.55–0.72)
SVM	0.71* (0.65–0.77)	0.68 (0.61–0.74)	0.70 (0.63–0.76)	0.71 (0.63–0.77)	0.64 (0.55–0.72)
RF	0.74 (0.68–0.80)	0.69 (0.62–0.75)	0.72 (0.65–0.78)	0.73 (0.65–0.79)	0.64 (0.55–0.72)
GBM	0.76 (0.70–0.82)	0.69 (0.62–0.75)	0.73 (0.66–0.79)	0.74 (0.66–0.80)	0.64 (0.55–0.72)
ANN	0.72 (0.66–0.78)	0.67 (0.60–0.74)	0.71 (0.64–0.77)	0.72 (0.64–0.78)	0.62 (0.53–0.70)

*p < 0.05 when compared with the AUC in GBM model through the DeLong test, while no statistical significance was observed between GBM model and RF or ANN model.

^#^The McNemar test showed that there was no significant difference in accuracy between any pair of models.

The DeLong test indicated that the GBM model had a significantly higher AUC compared with the LR and SVM models (both *p* < 0.05), but there were no significant differences when compared with the RF and ANN models (both *p* > 0.05). The McNemar test revealed no significant differences in accuracy between any of the model pairs (*p* > 0.05).

In the validation set, as shown in Table 4, the GBM model also performed well with an AUC of 0.75 (95% CI: 0.67–0.81), closely matching its performance in the testing set. The F1-score, sensitivity, accuracy and specificity of the GBM model in the validation set were 0.72 (95% CI: 0.63–0.79), 0.73 (95% CI: 0.63–0.81), 0.68 (95% CI: 0.61–0.74) and 0.63 (95% CI: 0.53–0.72), respectively.

**Table 4:** Performance metrics of different machine learning algorithms in the validation set (*n* = 195).

Algorithm	AUC	Accuracy	F1-score	Sensitivity	Specificity
LR	0.69 (0.61–0.76)	0.66 (0.59–0.72)	0.68 (0.60–0.74)	0.69 (0.59–0.77)	0.63 (0.53–0.72)
SVM	0.70 (0.62–0.77)	0.67 (0.60–0.73)	0.69 (0.61–0.75)	0.70 (0.60–0.78)	0.64 (0.54–0.73)
RF	0.73 (0.66–0.79)	0.68 (0.61–0.74)	0.71 (0.63–0.77)	0.72 (0.62–0.80)	0.64 (0.54–0.73)
GBM	0.75 (0.67–0.81)	0.68 (0.61–0.74)	0.72 (0.63–0.79)	0.73 (0.63–0.81)	0.63 (0.53–0.72)
ANN	0.71 (0.63–0.78)	0.66 (0.59–0.72)	0.70 (0.61–0.76)	0.71 (0.61–0.79)	0.61 (0.51–0.70)

### Explanation of prediction models

**Figure 1:**
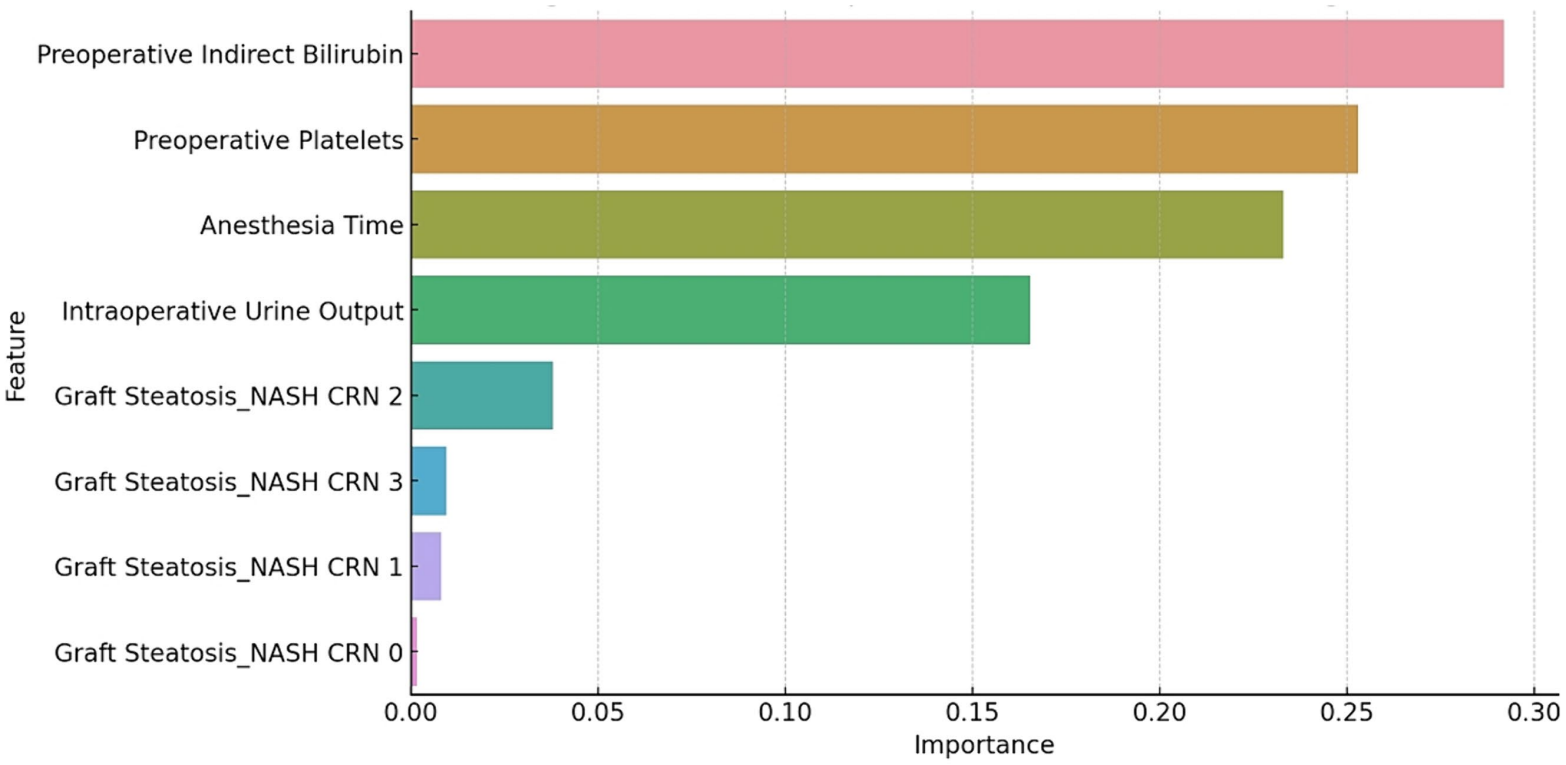
The SHAP summary plot for the GBM model. The plot shows the distribution of the SHAP values for each feature across all the cases. The features are ranked by their mean absolute SHAP value, which reflects their importance for the prediction.

The SHAP summary plot for the GBM model is illustrated in Figure 1. The top five features critical for predicting AKI following LT using DCD grafts are preoperative indirect bilirubin, intraoperative UO, anesthesia time, preoperative platelets and graft steatosis. Collectively, these features accounted for 57.8% of the total SHAP value.

**Figure 2:**
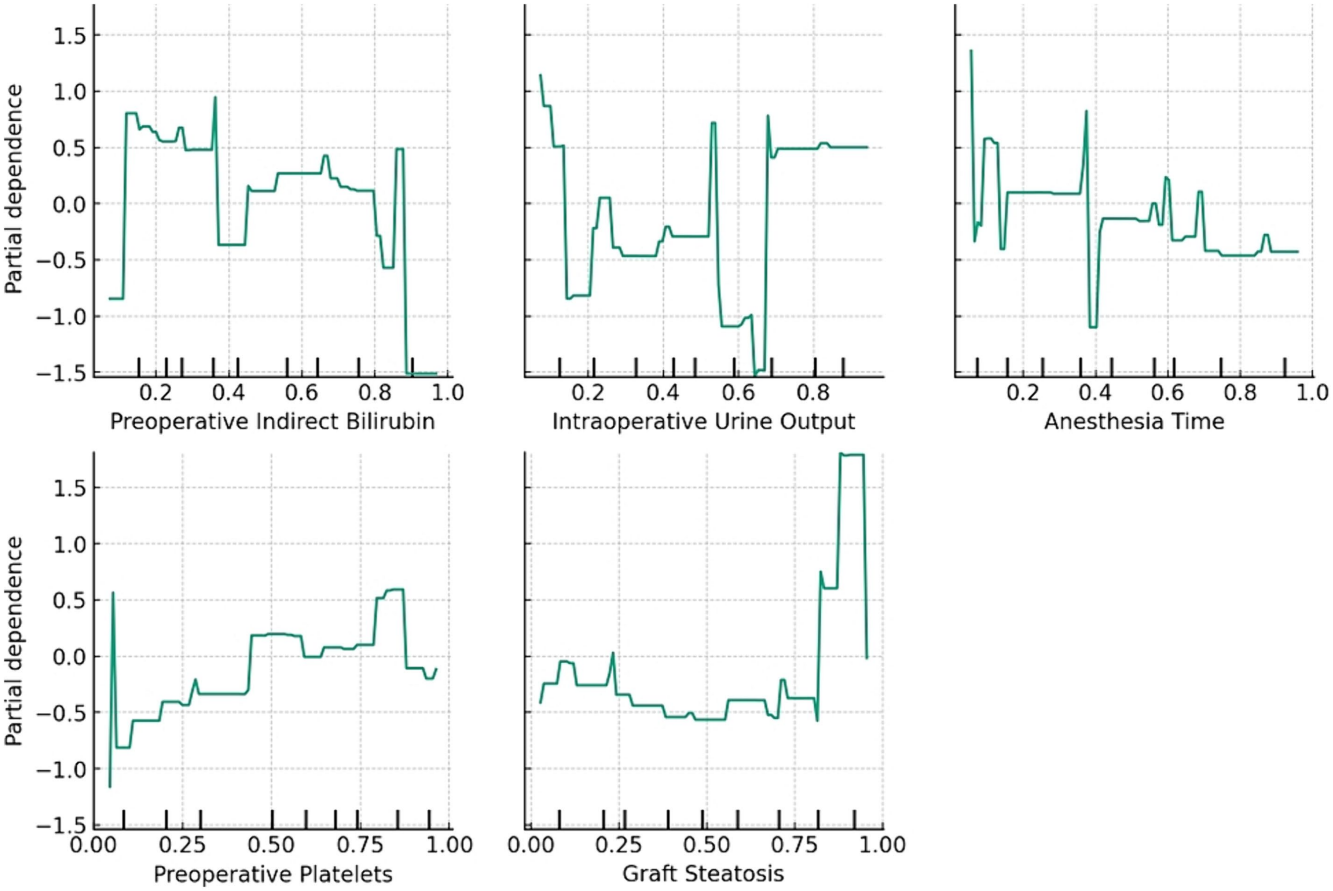
The relationship between the features and prediction outcome. The partial dependence plots show the average effect of the feature on the prediction.

The relationship between these features and the prediction outcomes is depicted in Figure 2, which presents the partial dependence plots for each feature. Preoperative indirect bilirubin had the highest mean absolute SHAP value (0.16), indicating its primary importance in predicting AKI post-LT with DCD grafts. Subsequently, the intraoperative UO (SHAP value of 0.13), anesthesia time (SHAP value of 0.10), preoperative platelets (SHAP value of 0.09) and graft steatosis (SHAP value of 0.08) sequentially demonstrated their significance in prediction. The partial dependence plot revealed a positive relationship between preoperative indirect bilirubin, anesthesia time and graft steatosis with the prediction outcome, whereas intraoperative UO and preoperative platelets displayed a negative and non-linear relationship.

We also compared the performance of the GBM model with that of a logistic regression model using the same predictors and outcomes. The logistic regression model achieved an AUC of 0.72 (95% CI: 0.66–0.78) in the testing set and 0.70 (95% CI: 0.62–0.77) in the validation set, which were notably lower than those of the GBM model (*p* < 0.05 by DeLong test). Furthermore, the logistic regression model exhibited lower accuracy, F1-score, sensitivity and specificity compared with the GBM model in both data sets.

## Discussion

In this study, we developed and validated a new prediction model for AKI following LT using DCD grafts based on machine-learning methods and clinical variables. We compared the performance of five machine learning algorithms and selected the GBM model as the most effective. The GBM model achieved robust AUC scores of 0.76 in the testing set and 0.75 in the validation set, and it also exhibited a high F1 score and sensitivity. Importantly, the GBM model identified five key features for predicting AKI following LT with DCD grafts: preoperative indirect bilirubin, intraoperative UO, anesthesia time, preoperative platelets and graft steatosis. The GBM model proved to be a reliable and interpretable tool, offering clinicians a means to identify high-risk patients and implement timely interventions to prevent or mitigate AKI.

Our research is the first to harness machine learning methods to predict AKI following LT using DCD grafts. Previous studies predominantly utilized conventional statistical methods, such as logistic regression or the Cox proportional hazards model, to develop prediction models for AKI post-LT; however, these were not specifically designed for DCD grafts, which possess distinct risk factors and pathophysiology compared with DBD grafts (16). Furthermore, traditional statistical methods face limitations, including assumptions of linearity and independence among predictors, difficulties in handling missing data and outliers and challenges in interpretability (10). Machine learning methods can surmount these issues, offering more accurate and robust predictions and providing deeper insights into the mechanisms driving AKI.

Upon comparing the five machine learning algorithms, we established that the GBM model excelled in terms of AUC, F1 score and sensitivity. The GBM model, an ensemble approach, enhances accuracy and robustness by boosting weak learners. It effectively manages non-linear and complex relationships among predictors, along with handling missing data and outliers. The GBM model has been widely utilized across various medical fields for diagnosis, prognosis, treatment and decision support (16). To the best of our knowledge, this study is the inaugural application of the GBM model in predicting AKI following LT using DCD grafts.

We also employed the SHAP method to elucidate predictions and pinpoint the crucial features of the GBM model. The SHAP method is innovative and robust, consistently providing locally accurate explanations for any machine-learning model. It assigns a value to each feature to represent its contribution to the prediction (17). Furthermore, the SHAP method offers various visual representations to illustrate the impact and distribution of each feature on prediction. While the SHAP method has been successfully applied to explain several machine learning models in medicine, including diagnosis, prognosis, treatment and decision support (18), to the best of our knowledge, this is the first study that utilizes the SHAP method to clarify the prediction model for AKI following LT with DCD grafts.

The GBM model identified five key features for predicting AKI post-LT with DCD grafts: preoperative indirect bilirubin, intraoperative UO, anesthesia time, preoperative platelets and graft steatosis. These features align with the existing literature and clinical understanding of the risk factors and pathophysiology of AKI in LT scenarios involving DCD grafts (19).

Preoperative indirect bilirubin serves as a marker of liver function and cholestasis, reflecting the severity of liver disease and the extent of portal hypertension. Elevated levels of preoperative indirect bilirubin may increase the risk of AKI due to hemodynamic instability, renal hypoperfusion, tubular injury and oxidative stress (20). High preoperative indirect bilirubin levels may also suggest compromised quality of the DCD graft, potentially heightening the risk of IRI and graft dysfunction post-LT (21).

Intraoperative UO acts as an indicator of renal function and perfusion, reflecting the effectiveness of fluid management and the likelihood of IRI. Reduced intraoperative UO can heighten the risk of AKI by inducing renal ischemia, tubular obstruction and acute tubular necrosis (22). Additionally, low intraoperative UO may signal an adverse outcome of LT, thereby increasing the risk of postoperative complications and mortality (23).

Anesthesia time serves as an indicator of the complexity and duration of LT, reflecting the extent of surgical trauma and blood loss. Extended anesthesia time may escalate the risk of AKI due to hemodynamic instability, inflammation, infection and electrolyte imbalance (24). Additionally, prolonged anesthesia time might suggest a challenging LT, which could increase the risk of graft dysfunction and rejection post-LT (25).

Preoperative platelets act as markers of coagulation and inflammation, indicative of the level of portal hypertension and the activation of the immune system. Reduced preoperative platelet counts may elevate the risk of AKI by promoting bleeding, infection and endothelial dysfunction (26). Furthermore, low preoperative platelets could signal a poor prognosis for LT, potentially leading to an increased risk of graft failure and sepsis post-LT (27).

Graft steatosis reflects the quality and viability of the DCD graft, indicating the extent of lipid accumulation and cellular damage in the liver. Higher grades of graft steatosis can amplify the risk of AKI by causing IRI, graft dysfunction, and bile duct complications post-LT (28). Moreover, substantial graft steatosis might also indicate a constrained donor pool and extended waiting times for LT (29).

We also noted that the severity of AKI varied among recipients who developed AKI post-LT with DCD grafts, predominantly with stage 2 or 3 AKI according to the KDIGO criteria. Previous studies have linked higher stages of AKI with poorer outcomes post-LT, including extended hospital stays, increased mortality and a heightened risk of developing CKD or ESRD (30). Thus, predicting not only the occurrence but also the severity of AKI post-LT with DCD grafts holds substantial clinical implications for risk stratification and management; however, our study did not incorporate the AKI stage as a predictor or outcome in the models, as our primary focus was on predicting the incidence of AKI post-LT using DCD grafts. Future research could investigate predicting the stages of AKI or the progression of AKI post-LT using DCD grafts, which would likely require more detailed and dynamic data on kidney function and injury biomarkers.

This study demonstrates that machine-learning-based methods, such as the GBM model, may offer advantages over traditional statistical approaches in predicting complex outcomes in clinical settings. Nevertheless, logistic regression models retain their merits, including simplicity, interpretability and familiarity among clinicians. The choice of the modeling approach should be tailored to the specific research question, the characteristics of the data and the intended application of the prediction model.

This study has several strengths. First, we utilized a large cohort of LT recipients who received DCD grafts from a single center, which enhanced the validity and reliability of our results; however, this may limit the generalizability to other settings or populations. Second, we employed machine learning methods to construct and compare prediction models for AKI following LT using DCD grafts, thereby improving the accuracy and robustness of our predictions. Third, we applied the SHAP method to explain and visualize the predictions and the crucial features of the optimal prediction model, increasing the interpretability and transparency of our results. Fourth, we validated the performance of the best prediction model in an independent cohort of LT recipients who received DCD grafts, confirming the reliability and applicability of our results.

The study also presents several limitations. First, we employed a retrospective and observational design, which might have introduced bias and confounding factors. Second, the binary outcome used for AKI may have overlooked variations in the severity and duration of AKI. Third, the exclusive use of clinical variables to build the prediction models could have excluded substantial biomarkers or imaging features. Fourth, we did not assess the clinical impact or cost-effectiveness of the prediction model, potentially limiting its adoption and implementation in practice. An additional limitation is the absence of external validation for our prediction model. Although we validated the performance of the GBM model in an independent cohort from our center, we did not test its accuracy or generalizability in other cohorts or settings. External validation is crucial to confirm the reliability and applicability of a prediction model, as it can evaluate the model’s performance across different populations, time periods, or geographic regions. Therefore, future studies should validate our GBM model in external cohorts of LT recipients who received DCD grafts, preferably from other centers or countries, to assess its robustness and transferability.

## Conclusion

In conclusion, we developed and validated a novel prediction model for AKI following LT using DCD grafts based on machine-learning methods and clinical variables. The GBM model exhibited the best performance in terms of AUC, F1 score and sensitivity. It identified five critical features for predicting AKI after LT using DCD grafts: preoperative indirect bilirubin, intraoperative UO, anesthesia time, preoperative platelets and graft steatosis. The GBM model appears to be a reliable and interpretable tool for predicting AKI following LT using DCD grafts. It may assist clinicians in identifying high-risk patients, enabling timely interventions to potentially prevent or mitigate AKI.

## Data availability statement

The original contributions presented in the study are included in the article/supplementary material, further inquiries can be directed to the corresponding authors.

## Ethics statement

The studies involving humans were approved by Beijing You’an Hospital of Capital Medical University (approval no. [2019]016). The studies were conducted in accordance with the local legislation and institutional requirements. The participants provided their written informed consent to participate in this study.

## Author contributions

H-XL: Investigation, Methodology, Project administration, Validation, Writing – original draft, Writing – review & editing. XW: Conceptualization, Methodology, Project administration, Validation, Writing – original draft, Writing – review & editing. M-MX: Formal analysis, Investigation, Methodology, Software, Validation, Writing – original draft, Writing – review & editing. YW: Conceptualization, Formal analysis, Methodology, Software, Validation, Writing – original draft, Writing – review & editing. ML: Formal analysis, Methodology, Software, Supervision, Visualization, Writing – original draft, Writing – review & editing. G-ML: Formal analysis, Investigation, Methodology, Supervision, Validation, Writing – original draft, Writing – review & editing. Q-HM: Conceptualization, Formal analysis, Methodology, Project administration, Writing – original draft, Writing – review & editing.
